# Editorial: Biointerfacing 2D nanomaterials and engineered heterostructures, volume II

**DOI:** 10.3389/fbioe.2024.1542498

**Published:** 2024-12-20

**Authors:** Alfredo Maria Gravagnuolo, Alessandro Martucci, Eden Morales-Naváez

**Affiliations:** ^1^ Independent Researcher, Nanobiotechnology Hub, Cambridge, United Kingdom; ^2^ Department of Industrial Engineering, University of Padova and INSTM, Padova, Italy; ^3^ Biophotonic Nanosensors Laboratory, Centro de Física Aplicada y TecnologíaAvanzada (CFATA), Universidad Nacional Autónoma de México (UNAM), Querétaro, Mexico

**Keywords:** graphene, MoS_2_, two-dimensional materials, nanocomposites, neural interfaces, cancer theragnostics, phototherapy, photoelectrochemical sensors

## 1 Introduction and aims

What does the future hold for two-dimensional (2D) materials? The future is the combination of biotechnology and nanotechnology. The potential applications of nanotechnology in the areas of healthcare and biomedicine are endless. Biological interfacing of 2D materials is a key step towards this paradigm.

Graphene is the first 2D material isolated, studied and produced of the history, although Sir. Andre Geim acknowledged some early ideas belonging to graphene “pre-history” (Geim, 2012). Today, after the 20th anniversary of its first characterization, graphene technology is not in its infancy any longer. The impact of research on the innovations, industrialization and commercialization is tangible right now, as proven by the variety of successful patents, commercial spin-offs and start-ups, and products. Some recently published and upcoming ISO standards, (ISO/TR 19733, 2019; ISO/TS 21356-1, 2021; ISO/TS 80004-13, 2024; ISO/AWI TS 21356-2, 2024; ISO/DTS 23359, 2024) are designed to boost the deployment of graphene technology. This is particularly necessary to introduce graphene in the highly regulated markets of products that come in direct contact with human, and to assess the environmental impact of generated waste. Standardized production processes, systematic structural assessment of graphene forms, and specific nomenclature for each member of the graphene family, will in turn facilitate the assessment of structure-properties, structure-safety relationships and market approvals by regulatory agencies.

In this perspective, recent human clinical trials, (ID NCT03659864 - University of Edinburgh, 2024; ID NCT06368310 David CoopeNorthern Care Alliance NHS Foundation Trust, 2024), intend to unleash a new era of graphene in healthcare applications (Andrews et al., 2024). In the current landscape of clinical translation, human clinical trials, which ensure rigorous toxicology investigation, performed in highly regulated setting, will build up robust and reliable toxicology data sets as fundaments for future uses in the main themes of biotechnology and medicine: (a) diagnostics, biosensing and imaging; (b) neural interfaces, prosthetic implants and other implantable devices; (c) cancer therapy and drug/gene delivery systems; (d) Tissue engineering and regenerative medicine (albeit this is a cross-functional theme with b and c); (e) antimicrobial and antiviral; (f) industrial bioprocesses, bioreactors and other nano-bio devices.

This editorial project started in 2019 with Vol I (hyperlink: https://www.frontiersin.org/research-topics/10687/biointerfacing-2d-nanomaterials-and-engineered-heterostructures/articles), aims to track advances in the most innovative fields of application of 2D nanomaterials, from graphene to the whole family of novel 2D materials, and their engineered heterostructures, which entails a deep understanding of phenomena happening upon biointerfacing.

## 2 Graphene neural interfaces

Graphene has proven ideal for neural interface applications, due to its remarkable electrical and mechanical properties, for stimulation and sensing, and high specific surface area for functionalization and cell bio interfacing. Ongoing clinical trials are investigating their functionality and side effects, to assess benefit-risk ratios associated with brain mapping in applications such as cancer brain surgery and treatment of Parkinson disease (ID NCT06368310 David CoopeNorthern Care Alliance NHS Foundation Trust, 2024).

In this article Research Topic, the comprehensive review titled “*Graphene-Based Nanomaterials (GBMs) for Peripheral Nerve Regeneration*” (Convertino et al.), considers the exploitation of GBMs biointerfaced, not only with the central nervous system (CNS), but above all with cells associated with peripheral nervous system (PNS), whose ever-increasing attention is being devoted. After describing the development of several graphene production methods, forms (e.g., 3D scaffolds, or conductive coatings) and formulations for nerve repair, the authors critically reviewed graphene interaction with peripheral neurons and, remarkably, highlighted the critical aspect of the local impact of non-neuronal cell alterations induced by the material on nerve regeneration. This no neuro-centric approach is quite broad-minded and highlights the need for a synergic contribution of many cell phenotypes to orchestrate a neuro-regenerative process, laying the foundational evidence of GBM safety and biocompatibility, which is a crucial aspect for clinical translation.

## 3 Two-dimensional materials and their multifunctional heterostructures in phototherapy and cancer medicine

Novel 2D materials, showing a full palette of physicochemical properties, are under the research spotlight. When assembled in Van der Waals vertical heterostructures, nanocomposites or other nanoassemblies not existing in the nature, they display exotic phenomena. Available in different forms such as powders, liquid dispersions, membranes and surface coatings, polymer nanocomposites and hydrogels, foams and aerogels, they virtually find application in any field. Biological interactions with biomolecules and living systems introduce the next functional dimensions. This article Research Topic highlights the significant potential of 2D layered materials and engineered heterostructures for therapy and diagnostics with three articles in the context of phototherapy and cancer theragnostics.

The review by Zhang et al. discusses the unique properties of these materials with recent trends in cancer diagnostics and therapy. The authors emphasize two key aspects. One, is the accurate screening of patients by means of capturing and detection of serum biomarkers, improved MRI/CT imaging capabilities and ultrasound-based diagnostics compared to traditional agents. Two, is the advancement in precision drug delivery including near-infrared radiation-based photothermal (PTT), photodynamic (PTD), sonodynamic, immuno and chemo-therapy. Ultimately, it underscores the promising role of 2D materials in cancer research and treatment, acknowledging the challenges associated with their development. Overall, it emphasizes the differences between 2D materials and other types of traditional nanoparticles (mainly spherical).

Feng et al. reported a groundbreaking photoelectrochemical (PEC) aptasensor, utilizing a double Z-scheme α-Fe_2_O_3_/MoS_2_/Bi_2_S_3_ ternary heterojunction, for the ultrasensitive detection of circulating tumor cells (CTCs). A key strength of this work is the successful synthesis of α-Fe_2_O_3_/MoS_2_/Bi_2_S_3_ nanocomposite via a step-by-step route. Photoproduced electron/hole transfer path was speculated by trapping experiments of reactive species. The α-Fe_2_O_3_/MoS_2_/Bi_2_S_3_-modified electrodes exhibited greatly enhanced photocurrent under visible light due to the double Z-scheme charge transfer process. All leveraged the unique performances of these materials to achieve high sensitivity and selectivity in CTC detection. The sensor’s broad linear range and low limit of detection are noteworthy, highlighting its applicability in clinical settings.

**FIGURE 1:**
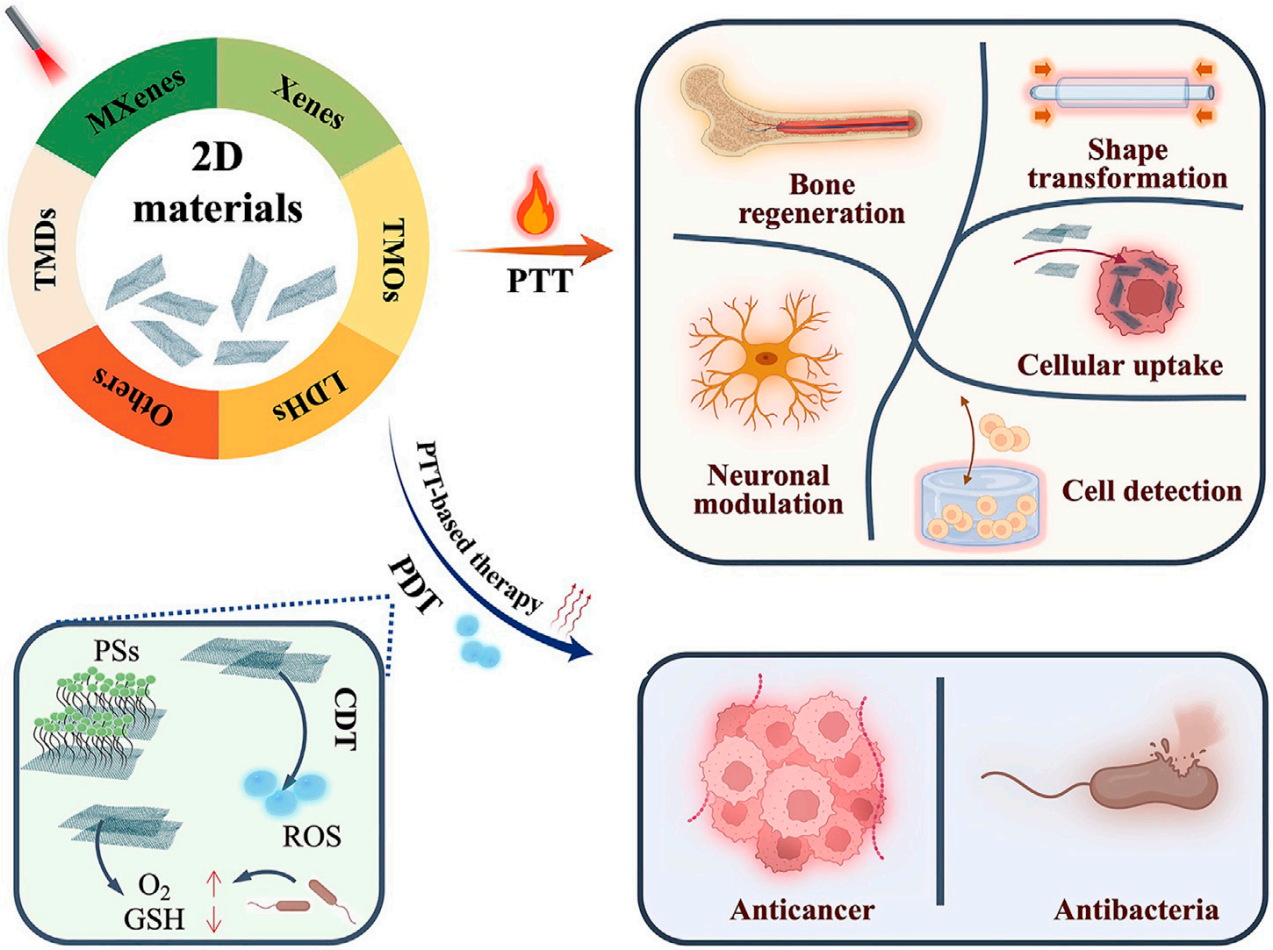
Biomedical applications of 2D materials beyond graphene in phototherapy, which generally refers to photothermal therapy (PTT) and photodynamic therapy (PDT). Reproduced with permission (Tan et al.).

The review by Tan et al. present the research progress of 2D materials in Phototherapy (Figure 1). New nanomaterials include nanoelemental nanosheets (Xenes), transition metal carbides, nitrides and carbonitrides (MXenes), transition metal dichalcogenides (TMDs), transition metal oxides (TMOs), layered double hydroxides (LDHs), metal-organic frameworks (MOFs), and Egyptian blue class (XCuSi_4_O_10_). The authors have meticulously summarized and discussed the recent advances, with several representative examples of anticancer, antibacterial, bone and neural regeneration, cell detection and drug delivery.

A lesson learned in the Vol II of this article Research Topic is that, whilst graphene has reached a mature stage towards clinical translation, it has also paved the way to a plethora of new 2D materials and heterostructures, which will follow in the upcoming years. Insisting on systematic physicochemical characterization, classification and toxicological evaluation is the key for the deployment of potentially endless technologies enabled by 2D materials and evaluation of risk-benefit ratio in each application.
